# COVID-19 in Adults With Hypertrophic Cardiomyopathy

**DOI:** 10.3389/fcvm.2021.745790

**Published:** 2021-11-09

**Authors:** Milla E. Arabadjian, Maria C. Reuter, Alexandra Stepanovic, Mark V. Sherrid, Daniele Massera

**Affiliations:** ^1^New York University Rory Meyers College of Nursing, New York, NY, United States; ^2^Hypertrophic Cardiomyopathy Program, Leon H. Charney Division of Cardiology, New York University Grossman School of Medicine, New York, NY, United States

**Keywords:** COVID-19, hypertrophic cardiomyopathy, outcomes, risk, obesity

## Abstract

**Background:** Individuals with cardiovascular disease are considered high risk for severe COVID-19. However, the clinical impact of COVID-19 in patients with hypertrophic cardiomyopathy (HCM) is unknown. The purpose of this study was to describe the clinical course and outcomes of COVID-19 in patients with HCM.

**Methods:** This retrospective observational study included adults with HCM and positive PCR/antibody test for SARS-CoV-2 at a large urban hospital system in the New York from January, 2020 to January, 2021.

**Results:** Seventy individuals were included, with a mean (SD) age of 60.1 (15.1) years, 39 (55.7%) of whom were male, and 42 (60%) white. Forty-five (65.3%) patients had obstructive HCM. Hypertension and obesity (BMI ≥ 30) were present in 45 (64.3%) and 37 (52.9%) patients, and the prevalence of atrial fibrillation, obstructive sleep apnea and diabetes was high. Common symptoms of COVID-19 were fever, cough, shortness of breath and fatigue, affecting 33 (47.1%), 33 (47.1%), 28 (40.0%), and 28 (40.0%) patients, respectively. Fourteen (20%) patients were hospitalized. The majority (45 [64.3%] patients) recovered without intervention. Two patients had non-fatal pulmonary embolisms, 1 had atrial fibrillation requiring electrical cardioversion and 1 had acute decompensated heart failure. Three (4.3%) patients required mechanical ventilation, two of whom died (case fatality rate 2.9%). A total of 15 (21.4%) patients were asymptomatic.

**Conclusions:** Our data suggest that in this diverse and high-risk group of patients with HCM, established risk factors for severe COVID-19, such as obesity, may be more important drivers of morbidity and mortality than the presence of HCM alone.

## Introduction

Since the beginning of the COVID-19 pandemic, over 200 million cases with over 4 million deaths worldwide have been recorded (1). Cardiovascular disease has emerged as risk factor for increased morbidity and mortality (2–4). Heart failure in particular was shown to be associated with worse outcomes (5). Hypertrophic cardiomyopathy is the most common inherited cardiac disorder and is characterized by cardiac hypertrophy, left ventricular outflow obstruction in the majority of cases, and diastolic dysfunction (6). We were particularly concerned about outcomes of patients with hypertrophic cardiomyopathy (HCM) due to reports of ACE2 gene upregulation in septal myectomy specimens (7) — the ACE2 receptor being the entry point for the SARS-CoV-2 virus. However, the impact of preexisting HCM on the clinical course of COVID-19 is currently unknown. This study aimed to examine the outcomes of COVID-19 in patients with HCM.

## Materials and Methods

To address this question, we performed a retrospective cohort study of consecutive adult patients, age ≥18 years with HCM who underwent testing for COVID-19 PCR or antibodies at NYU Langone Health, a large urban healthcare system in the New York City area with hospitals in Manhattan, Brooklyn, and Nassau County between January 1, 2020 and January 6, 2021. Individuals were included in the study if they had either positive SARS-CoV-2 PCR or antibody testing. The study was approved by the NYU Grossman School of Medicine's Institutional Review Board and informed consent was waived.

Imaging studies were reviewed by one cardiologist (D.M.) to confirm HCM diagnosis by current guidelines (6) and to identify HCM structural characteristics including distribution of left ventricular hypertrophy (LVH), systolic anterior motion (SAM), left ventricular outflow tract (LVOT) obstruction, mid-ventricular obstruction, apical aneurysm, and mitral annular calcification (MAC). Clinical information was recorded, including medical therapy, history of septal myectomy or alcohol septal ablation, comorbid conditions, COVID-19 presenting symptoms, treatment, hospitalizations, and outcomes. Statistical analysis was performed using STATA 16 (StataCorp LLC, College Station, TX) (8). Data were expressed as means and standard deviation (SD) or medians (interquartile range) for continuous variables, and proportions for categorical variables, as appropriate. The unpaired *t*-test or the Mann Whitney U test were used to compare continuous variables, as appropriate, and the chi-squared or Fisher's exact test for categorical variables. A *p* < 0.05 was considered significant.

## Results

### Demographics and HCM Characteristics

**Figure 1:**
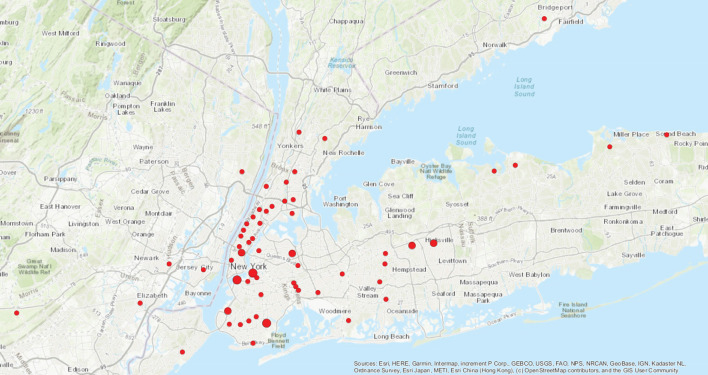
Distribution of patients by zip code. 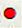
One patient per zip code. 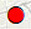
Two patients per zip code. 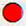
Three patients per zip code.

A total of 70 patients with HCM had positive COVID-19 testing, 58 (82.9%) of whom by PCR and 12 (17.1%) only by antibody testing. Median (IQR) age was 62 (51–71) years, 39 (55.7%) were male. There were 42 (60%) white, 19 (27%) Black, and 5 (7%) Asian individuals, of whom 10 (14%) were of Hispanic ethnicity. Individuals resided in the NYC metropolitan tri-state area (including Westchester County, Nassau County, Suffolk County, New Jersey and Connecticut) (Figure 1).

The majority of patients, 45 (64%), in this sample had obstructive HCM, defined as ≥30 mmHg LVOT or mid-ventricular gradient at rest or with provocation. Apical aneurysm was present in five (7.1%) patients, all of whom had a mid-systolic Doppler signal void (9). Asymmetric septal hypertrophy (ASH) was present in 60 (85.7%) individuals, Mean (SD) left ventricular ejection fraction was 69.6 (5.6) % and mean maximal LV wall thickness was 17.6 (4.2) mm. Fifteen patients (21.7%) had an implantable cardioverter defibrillator and one patient had a permanent pacemaker. Most patients were on standard HCM specific medical therapy including beta blockers and calcium channel blockers (Table 1). Disopyramide was used in 11 (15.9%) patients and 15 (21.4%) had a prior history of surgical septal myectomy; none had alcohol septal ablation. A total of 26 (37.1%) individuals were taking QT-prolonging drugs.

**Table 1:** Demographics and baseline HCM characteristics.

	**Total** ***N* = 70**	**Hospitalized** ***N*=14**	**Non-hospitalized *N* = 56**	***p*-value**
Age, median (IQR), years	62 (51–71)	64.5 (55–78)	61.5 (48.5–70)	0.155
Male sex, *N* (%)	39 (55.7)	6(42.9)	33 (58.9)	0.279
Race, *N* (%) White Black/African American Asian	42 (60.0) 19 (27.1) 5 (7.1)	8 (57.1) 6 (42.9) 0 (0)	34 (60.7) 13 (23.2) 5 (8.9)	0.569
Ethnicity, *N* (%) Hispanic	10 (14.3)	2 (14.3)	8 (14.3)	0.967
LV ejection fraction, median (IQR), %	70 (65–75)	72.5 (65–75)	70 (65–75)	0.208
Maximum LV thickness, median (IQR), mm	17 (15–20)	18 (16–20)	17 (15–20)	0.370
Type HCM, *N* (%) Non-obstructive Obstructive	23 (32.9) 47 (67.1)	6 (42.9) 8 (57.1)	17 (30.4) 39 (69.6)	0.373
Distribution of LV hypertrophy, *N* (%) Asymmetric septal hypertrophy Concentric hypertrophy Apical hypertrophy	60 (85.7) 4 (5.8) 15 (21.4)	11 (78.6) 0 (0) 2 (14.3)	48 (85.7) 4 (7.1) 13 (23.2)	0.608
Apical aneurysm Pulmonary hypertension	5 (7.1) 4 (5.7)	1 (7.1) 3 (21.4)	4 (7.1) 1 (1.8)	0.684 0.023
≥ moderate mitral regurgitation, *N* (%)	4 (5.7)	0 (0)	4 (7.1)	0.401
≥ moderate aortic stenosis, *N* (%)	1 (1.4)	0 (0)	1 (1.8)	0.200
History of surgical septal myectomy, *N* (%)	15 (21.4)	1 (7.1)	14 (25)	0.135
Beta blocker, *N* (%) Calcium channel blocker, *N* (%) Disopyramide, *N* (%) ACE/ARBs, *N* (%) Amlodipine, *N* (%) Diuretics, *N* (%) Anti-coagulant therapy, *N* (%) Anti-platelet agents, *N* (%)	58 (82.9) 16 (22.9) 11 (15.7) 14 (20.0) 8 (11.4) 22 (31.4) 19 (27.1) 22 (31.4)	11 (78.6) 5 (35.7) 4 (28.6) 2 (14.3) 3 (21.4) 5 (35.7) 7 (50) 4 (28.6)	47 (83.9) 11 (20) 7 (12.5) 12 (21.4) 5 (8.9) 17 (30.4) 12 (21.4) 18 (32.1)	0.634 0.214 0.143 0.494 0.193 0.699 0.032 0.506
Implantable cardioverter defibrillator, N (%)	15 (21.7)	2 (14.3)	13 (23.2)	0.374

### Co-morbidities

The overall burden of co-morbidities was high (Table 2). The most common co-morbidities were hypertension in 45 (64.3%), obesity (BMI>30 kg/m^2^) in 37 (52.9%), atrial fibrillation in 23 (32.3%), coronary artery disease in 17 (24.3%), obstructive sleep apnea in 15 (21.4%) and diabetes in 15 (21.4%) patients. Individuals in this sample had a median (IQR) of three (2–5) co-morbidities in addition to HCM. There were no significant differences in co-morbidities between racial/ethnic groups and between men and women (data not shown).

**Table 2:** Co-morbidities.

	**Total** ***N* = 70**	**Hospitalized** ***N* = 14**	**Non-hospitalized *N* = 56**	***p*-value**
Hypertension, *N* (%)	45 (64.3)	11 (78.6)	34 (60.7)	0.212
Hyperlipidemia, *N* (%)	41 (58.6)	7 (50)	34 (60.7)	0.467
Coronary artery disease, *N* (%)	17 (24.3)	4 (28.6)	13 (23.2)	0.676
Atrial fibrillation, *N* (%)	23 (32.3)	5 (35.7)	18 (32.1)	0.799
Stroke or transient ischemic attack, *N* (%)	5 (7.1)	1 (7.1)	4 (7.1)	0.684
BMI, mean (SD), kg/m^2^	31.6 (6.0)	32.1 (1.6)	31.2 (0.81)	0.805
BMI > 30, *N* (%)	37 (52.9)	9 (64.3)	28 (50.0)	0.338
DM, *N* (%) Insulin dependent Non-insulin dependent	3 (4.3) 12 (14.3)	1 (7.1) 3 (21.4)	2 (3.4) 9 (16.1)	0.591
Liver disease, *N* (%) Hepatitis C Steatosis	2 (2.9) 3 (4.3)	0 (0) 2 (14.3)	2 (3.6) 1 (1.8)	0.300
Chronic kidney disease, *N* (%)	7 (10.0)	3 (21.4)	4 (7.3)	0.142
Asthma or COPD, *N* (%)	9 (12.9)	3 (21.4)	6 (10.7)	0.253
Obstructive sleep apnea, *N* (%)	15 (21.4)	3 (21.4)	12 (21.4)	0.626
Cancer – active, *N* (%)	1 (1.4)	0 (0)	1 (1.8)	0.800
HIV, *N* (%)	1 (1.4)	0 (0)	1 (1.8)	0.800
Number of cardiac co-morbidities, median (IQR)	2 (1–3)	2 (1–3)	2 (1–3)	0.477
Number of total co-morbidities, median (IQR)	3 (2–5)	3.5 (2–5)	3 (2–5)	0.296

### COVID-19 Course and Outcomes

Fifteen individuals (24.3%) in this cohort were asymptomatic. The most common symptoms included fever in 33 (47.1%), cough in 33 (47.1%), shortness of breath in 28 (40.0%) and fatigue in 28 (40.0%) patients (Table 3). The majority of patients, 45 (64.3%), did not seek or require medical interventions. Medical therapy included the use of antibiotics and steroids. Azithromycin was given to five (7.1%) patients and hydroxychloroquine to six (8.6%). Fourteen (20.0%) patients were hospitalized. There were no significant differences in demographics, HCM characteristics, or co-morbidity profile between the hospitalized and the non-hospitalized group. However, there were differences in presenting symptoms between the groups, as expected. The majority of hospitalized patients presented with shortness of breath, cough and fever than the non-hospitalized patients, 13 (92.9%) vs. 15 (26.8%), *p* < 0.001, 10 (71.4%) vs. 23 (41%), *p* = 0.042, and 10 (71.4%) vs. 23 (41%), *p* = 0.042, respectively. Furthermore, treatment differed between the hospitalized and non-hospitalized patients, with hospitalized patients utilizing more antibiotic therapy. Four of the hospitalized individuals required intensive care and three required intubation and mechanical ventilation. Two patients (2.9%) died during hospital admission, one woman and one man, both non-Hispanic whites, ages 69 and 71 years, and both obese, with BMI of 38 and 35.3 kg/m^2^. The female patient had non-obstructive HCM (maximal wall thickness 23 mm), hypertension, hyperlipidemia, non-obstructive coronary artery disease, non-insulin dependent diabetes, COPD, liver steatosis, paroxysmal atrial fibrillation, chronic renal disease, and an implantable cardiac defibrillator for primary prevention of sudden cardiac death. The male patient had obstructive HCM with SAM, LVOT obstruction with a peak LVOT gradient at rest of 108 mmHg, moderate aortic valve stenosis and obstructive sleep apnea. In addition, two (2.9%) patients presented with non-fatal pulmonary embolisms, one of whom was also found to have a tibial deep vein thrombosis and presented with atrial fibrillation requiring electrical cardioversion. One patient (1.4%) presented with acute decompensated heart failure requiring intravenous diuretics.

**Table 3:** COVID-19 symptoms, treatment, outcomes.

	**Total** ***N* = 70**	**Hospitalized** ***N* = 14**	**Non-hospitalized** ***N* = 56**	***p*-value**
COVID-19 diagnostic testing, *N* (%) Positive SARS-CoV-2 PCR Positive SARS-CoV-2 IgG antibodies only	58 (82.9) 12 (17.1)	14 (100) 0 (0)	44 (78.6) 12 (21.4)	0.057
Presenting symptoms, *N* (%) None Fever Cough Shortness of breath Fatigue GI distress Loss of smell/taste	15 (21.4) 33 (47.1) 33 (47.1) 28 (40.0) 28 (40.0) 6 (8.6) 11 (15.7)	0 (0) 10 (71.4) 10 (71.4) 13 (92.9) 3 (21.4) 2 (14.3) 0 (0)	15 (26.8) 23 (41) 23 (41) 15 (26.8) 25 (44.6) 2 (7.1) 11 (19.6)	0.023 0.042 0.042 <0.001 0.113 0.345 0.069
COVID-19 therapy, *N* (%) None/supportive Azithromycin Hydroxychloroquine Non-macrolide antibiotics Steroids Remdesivir	45 (64.3) 5 (7.1) 6 (8.6) 6 (8.6) 7 (10.0) 1 (1.4)	5 (35.7) 4 (28.6) 5 (35.7) 3 (21.4) 4 (28.6) 1 (7.1)	40 (71.4) 1 (1.8) 1 (1.8) 3 (5.4) 3 (5.4) 0 (0)	0.045 0.005 0.001 0.090 0.026 0.200
COVID-19-associated complications in hospitalized patients, No. (%)				
Pulmonary embolism, *N* (%) Deep vein thrombosis, *N* (%) Thrombocytopenia, *N* (%) Atrial fibrillation requiring cardioversion, *N* (%) Acute decompensated heart failure	2 (2.9) 1 (1.4) 1 (1.4) 1 (1.4) 1 (1.4)	
ICU hospitalization, *N* (%)	4 (5.7)	
Intubated, *N* (%)	3 (4.3)	
Deceased, *N* (%)	2 (2.9)	

## Discussion

This study is the first to examine the clinical course and outcomes of COVID-19 in patients with HCM, a significant proportion of whom had prior septal myectomy surgery and implantable cardioverter defibrillators. The hospital admission rate was high at 20%.The case fatality rate in this sample was similar to the general population (1). Both individuals who died had multiple co-morbid conditions associated with higher morbidity and mortality [2, 3.] Among hospitalized patients, the distribution of non-obstructive and obstructive HCM patients mirrors the distribution in unselected HCM cohorts (10). There were no significant differences in demographics, HCM characteristics, or COVID-19 risk factors between the hospitalized and not hospitalized group. One reason for this may be that only four patients required an ICU level of care. Moreover, a majority of the non-ICU hospitalized patients were treated with supportive care. It is likely that most were admitted for observation and monitoring of possible COVID-19 related deterioration, given that the presenting COVID-19 related symptom of the vast majority of hospitalized patients was shortness of breath. The overall low numbers of seriously ill HCM patients with COVID-19 in the sample preclude an adequate statistical analysis on risk profiles. Two patients presented with non-fatal pulmonary embolisms, a known complication of COVID-19 (11). Furthermore, even though one patient presented with acute decompensated heart failure, this is not uncommon in the setting of acute illness (12).

Prior reports have noted that there is ACE2 receptor upregulation in HCM tissue specimens (7), and ACE2 is the entry point of SARS-Cov-2. One small study examined cardiac samples from individuals with dilated cardiomyopathy, hypertrophic cardiomyopathy and healthy controls, which also supported upregulation of ACE2 in HCM tissue, but did not observe a difference in ACE2 expression between HCM patients taking ACE inhibitor medicines and those who did not (13). However, the clinical impact of this upregulation in HCM is unclear. Our study is the first to examine the clinical impact of COVID-19 on HCM patients in real world conditions. Our data suggest that HCM may not in itself contribute to worse clinical outcomes from COVID-19, above other established risk factors, such as age and obesity. Further studies in larger cohorts of patients with HCM and COVID-19 are needed.

Limitations of this study include the possibility of selection bias, underreporting and asymptomatic infections leading to a high admission rate. Furthermore, the relatively small sample size may limit the generalization of results. However, our cohort is representative of a diverse population. Last, as patients with HCM were deemed high risk for COVID-19 complications along with other types of cardiovascular disease, this awareness may have led to strict adherence with social distancing recommendations, which may have been the cause of a lower infection rate.

In conclusion, our data suggest that HCM in itself does not carry a higher risk of COVID-19 disease severity and complications. Established risk factors for severe COVID-19, such as age and obesity may be more influential.

## Data Availability Statement

The raw data supporting the conclusions of this article will be made available by the authors upon reasonable request.

## Ethics Statement

The studies involving human participants were reviewed and approved by NYU Langone Health Institutional Review Board. Written informed consent for participation was not required for this study in accordance with the national legislation and the institutional requirements.

## Author Contributions

MA: conceptualization, methodology, formal analysis, investigation, data curation, and writing—original draft. MR: investigation, writing—review, and editing. AS: investigation. MS: writing—review and editing. DM: conceptualization, methodology, data curation, supervision, and writing—original draft. All authors on this manuscript have fulfilled the criteria for authorship as set forth by the ICMJE guidelines.

## Funding

This study was supported by internal funds from the Leon H. Charney Division of Cardiology, Department of Medicine, NYU Langone Health.

## Conflict of Interest

MA reports modest advisory board fees from MyoKardia, Inc. MR reports modest advisory board fees from MyoKardia, Inc. MS reports consulting fees from Celltrion. DM reports consulting fees from Bristol Myers Squibb. The remaining author declares that the research was conducted in the absence of any commercial or financial relationships that could be construed as a potential conflict of interest.

## Publisher's Note

All claims expressed in this article are solely those of the authors and do not necessarily represent those of their affiliated organizations, or those of the publisher, the editors and the reviewers. Any product that may be evaluated in this article, or claim that may be made by its manufacturer, is not guaranteed or endorsed by the publisher.

## References

[1] WHO. WHO Coronavirus (Covid-19) Dashboard. (2021). Available online at: https://covid19.who.int/. (Accessed April 22, 2020)

[2] Zhou F Yu T Du R Fan G Liu Y Liu Z. Clinical course and risk factors for mortality of adult inpatients with COVID-19 in Wuhan, China: a retrospective cohort study. Lancet. (2020) 395:1054–62. 10.1016/S0140-6736(20)30566-3 32171076 PMC7270627

[3] Parohan M Yaghoubi S Seraji A Javanbakht MH Sarraf P Djalali M. Risk factors for mortality in patients with coronavirus disease 2019 (COVID-19) infection: a systematic review and meta-analysis of observational studies. Aging Male. (2020) 23:1416–24. 1–9. 10.1101/2020.04.09.20056291 32508193

[4] Katz MH. Regardless of age, obesity and hypertension increase risks with COVID-19. JAMA Intern Med. (2021) 181:381. 10.1001/jamainternmed.2020.5415 32902563

[5] Alvarez-Garcia J Lee S Gupta A Cagliostro M Joshi AA Rivas-Lasarte M. Prognostic impact of prior heart failure in patients hospitalized with COVID-19. J Am Coll Cardiol. (2020) 76:2334–48. 10.1016/j.jacc.2020.09.549 33129663 PMC7598769

[6] Ommen SR Mital S Burke MA Day SM Deswal A Elliott P. 2020 AHA/ACC guideline for the diagnosis and treatment of patients with hypertrophic cardiomyopathy: executive summary: a report of the American college of cardiology/American heart association joint committee on clinical practice guidelines. J Am Coll Cardiol. (2020) 76:3022–55. 10.1016/j.jacc.2020.08.044 33229115

[7] Bos JM Hebl VB Oberg AL Sun Z Herman DS Teekakirikul P. Marked up-regulation of ACE2 in hearts of patients with obstructive hypertrophic cardiomyopathy: implications for SARS-CoV-2–mediated COVID-19. Mayo Clin Proc. (2020) 95:1354–68. 10.1016/j.mayocp.2020.04.028 32448590 PMC7186205

[8] StataCorp. Stata Statistical Software: Release 16. College Station, TX: StataCorp LLC (2019).

[9] Po JRF Kim B Aslam F Arabadjian M Winson G Cantales D. Doppler systolic signal void in hypertrophic cardiomyopathy: apical aneurysm and severe obstruction without elevated intraventricular velocities. J Am Society of Echocardiogr. (2015) 28:1462–73. 10.1016/j.echo.2015.08.015 26422555

[10] Maron BJ. Clinical course and management of hypertrophic cardiomyopathy. N Engl J Med. (2018) 379:655–68. 10.1056/NEJMra1710575 30110588

[11] Bikdeli B Madhavan MV Jimenez D Chuich T Dreyfus I Driggin E. COVID-19 and thrombotic or thromboembolic disease: implications for prevention, antithrombotic therapy, and follow-up: JACC state-of-the-art review. J Am Coll Cardiol. (2020) 75:2950–73. 10.1016/j.jacc.2020.04.031 32311448 PMC7164881

[12] Rey JR Caro-Codón J Rosillo SO Iniesta ÁM Castrejón-Castrejón S Marco-Clement I. Heart failure in COVID-19 patients: prevalence, incidence and prognostic implications. Eur J Heart Fail. (2020) 22:2205–15. 10.1002/ejhf.1990 32833283 PMC7461427

[13] Tucker NR Chaffin M Bedi KC Jr Papangeli I Akkad AD Arduini A. Myocyte-specific upregulation of ACE2 in cardiovascular disease: implications for SARS-CoV-2–mediated myocarditis. Circulation. (2020) 142:708–10. 10.1161/CIRCULATIONAHA.120.047911 32795091 PMC7424896

